# Oral Health as a Marker of Social Progress

**DOI:** 10.1177/00220345251324120

**Published:** 2025-04-22

**Authors:** S. Listl, T. Bärnighausen

**Affiliations:** 1 Heidelberg University Hospital, Heidelberg Institute of Global Health, Section for Oral Health, Germany

**Keywords:** human development, outcome measures, social accountability, quality of life, economic growth, dentistry

## Abstract

Society strives to improve the quality of life and well-being of its members. While a broad range of social, economic, political, health, and environmental dimensions have been considered to contribute to human development, the perception of what constitutes social progress has been evolving over time. This article provides an overview of widely used social progress indices and their previous lack of oral health metrics. The article further discusses how information on oral health can portray dimensions of social progress over and above conventional markers of social progress. The article identifies unique characteristics of oral health, which qualify it as a relevant marker of social progress. To optimize people’s life and well-being, we argue that concise oral health metrics (tentative items: number of natural teeth; global oral health rating; unmet oral health needs) can put oral health forward as a pertinent and practical marker of social progress. The World Health Organization’s global strategy and action plan on oral health 2023–2030 offers a unique window of opportunity to enhance the uptake of oral health as a routine and central marker of social progress. The feasibility of implementing oral health as a marker of social progress has recently been exemplified by the European Commission’s inclusion of an oral health–specific indicator in the 2024 edition of the EU regional Social Progress Index. Future research is encouraged to demonstrate the worldwide viability of concise and easy-to-use oral health metrics for inclusion in indices to measure social progress. Oral health is critical to ensure social progress and human development.

## The Relevance of Measuring Social Progress

As society strives to improve the quality of life and well-being of its members (UN General Assembly [UNGA] 1970), the notion of social progress has been characterized as follows (Estes et al. 2023):

Social progress refers to progressive advancements over time in the capacity of nations to satisfy at least the basic social, material, and spiritual needs of their populations. A well-established construct in the literatures of sociology . . . , political science . . . , economics . . . , philosophy, and comparative religion, social progress embraces both the means required for realizing fuller levels of individual and collective development as well as the ends that are to result from that development (e.g., improved educational status, improved housing and sanitation conditions, better health, increased spirituality). Thus, social progress is both an intrinsic and instrumental value in social development and, as such, reflects the processes and outcomes associated with positive changes that take place on an intentional basis in the lives of people over time.

Measuring social progress is crucial for understanding and improving the well-being of societies beyond economic growth (United Nations Development Programme 1990; UN Economic and Social Council 2024). By focusing on human needs and capabilities, sensible metrics for social progress help ensure that development is inclusive and holistic, promoting a higher quality of life for all (Sen 1999). Suitable measures of social progress offer reliable insights into health, education, equity, and environmental quality, which are essential for human development (Porter et al. 2015). Such measures also allow comparisons across countries and regions, fostering global accountability and cooperation (Fehder et al. 2018). Neglects in measuring social progress can pose risks of jeopardizing people’s quality of life and well-being. While oral health has previously been neglected in the broader global policy landscape, the World Health Organization’s (WHO’s) global strategy and action plan on oral health 2023–2030 emphasize oral health as an integral part of overall health (WHO 2024). Achieving the highest attainable standard of oral health is considered a fundamental right of every human being (WHO 2024).

After providing an overview of existing social progress indices, this article discusses why and how oral health can portray important dimensions of social progress over and above conventional health metrics. In light of the previous absence of oral health within social progress indices, the article first and foremost seeks to establish the foundational relevance of including oral health in social progress indices. In addition, the article discusses relevant aspects for operationalizing oral health indicators in terms of their measurement properties and in terms of suitable data sources to populate such social progress indices with oral health information.

## Existing Social Progress Indices and Their Lack of Oral Health Metrics

Several indices have been developed to provide a comprehensive assessment of social progress, considering various dimensions such as health, education, safety, environment, and personal freedom. While a detailed discussion of all existing indices to measure social progress is beyond the scope of this article, it is pertinent to consider commonly used indices and their lack of oral health metrics (see Table 1).

**Table 1.** Lack of Oral Health Metrics in Select Social Progress Indices.

Index	Dimensions	Health Metrics	Oral Health Metrics^a^
Human Development Index	• Health • Education • Standard of Living	• Life expectancy at birth	No
Social Progress Index	• Basic Human Needs • Foundations of Well-being • Opportunity	• Life expectancy • Prevalence of communicable and noncommunicable diseases • Child mortality • Access to essential services	Previously no, but the European Commission introduced unmet dental needs as a new indicator in its 2024 edition of the EU regional Social Progress Index (EU-SPI 2.0)
Multidimensional Poverty Index	• Health • Education • Living Standards	• Nutrition • Child mortality	No
OECD Better Life Index	11 dimensions	• Objective measures: life expectancy, mortality rates • Subjective measures: self-reported health	No
UN Sustainable Development Goals (SDGs)	17 SDGs	SDG 3 - Good Health and Well-being: • Maternal mortality reduction • Neonatal and child mortality reduction • Infectious diseases reduction • Noncommunicable diseases reduction • Substance abuse reduction • Road traffic reduction • Universal access to sexual and reproductive health care services • Universal health coverage (UHC) • Environmental health improvement	No, but WHO strategy and action plan on oral health are aligned with the UN SDGs, particularly SDG 3 and its UHC target

OECD, Organization for Economic Cooperation and Development; UN, United Nations; WHO, World Health Organization;

a Oral health metrics explicitly mentioned: yes or no.

### Human Development Index (HDI)

The HDI is one of the most widely recognized frameworks for assessing social progress. Developed by the United Nations Development Programme (United Nations Development Programme 1990), the HDI measures a country’s average achievements in 3 fundamental dimensions of human development:

- Health: Measured by life expectancy at birth
- Education: Measured by the average number of years of schooling for adults aged 25 y or older and the expected years of schooling for children entering school
- Standard of Living: Measured by gross national income per capita, adjusted for purchasing power parity

The HDI is a composite index that assigns equal weight to each of the 3 dimensions described above. It ranges from 0 to 1, with higher values indicating higher levels of human development. The HDI has been widely adopted due to its simplicity and focus on essential aspects of human well-being beyond purely economic measures such as gross domestic product (GDP).

### Social Progress Index (SPI)

The SPI was developed by Social Progress Imperative, an international nonprofit organization, to provide a more nuanced view of social progress that goes beyond economic indicators (Porter et al. 2015). The SPI measures social progress across 3 broad dimensions:

- Basic Human Needs: Includes indicators such as nutrition and basic medical care, water and sanitation, shelter, and personal safety
- Foundations of Well-being: Encompasses access to basic knowledge, access to information and communications, health and wellness, and environmental quality
- Opportunity: Covers personal rights, personal freedom and choice, inclusiveness, and access to advanced education

Each of the 3 SPI dimensions is further broken down into multiple components. Health-related indicators are typically part of “Basic Human Needs” (e.g., child mortality, access to basic medical care) or “Foundations of Well-being” (e.g., life expectancy, prevalence of communicable and noncommunicable diseases). Unlike the HDI, the SPI does not include economic measures, focusing instead on the outcomes of social development efforts. The SPI emphasizes outcomes over inputs, highlighting disparities in health and well-being that economic data might overlook. For instance, 2 countries with similar GDP per capita can have vastly different SPI scores if one has better health care access and outcomes. Therefore, the SPI is a valuable tool for policy makers to identify and address social and health disparities, prioritizing human well-being alongside economic development (Porter et al. 2015). Contrary to the vast and long-standing worldwide lack of oral health–related indicators in social progress indices, the European Commission recently introduced “unmet dental needs” as a new indicator in its 2024 edition of the EU regional Social Progress Index (EU-SPI 2.0; European Commission 2024).

### Multidimensional Poverty Index (MPI)

The MPI was developed by the Oxford Poverty and Human Development Initiative and the United Nations Development Programme in 2010 to address the limitations of traditional poverty measures that focus solely on income (Alkire and Santos 2010; United Nations Development Programme 2020). The MPI assesses poverty through the following dimensions:

- Health: Indicators such as nutrition and child mortality
- Education: Measured by years of schooling and school attendance
- Living Standards: Includes indicators such as access to electricity, sanitation, safe drinking water, housing quality, and assets

Each dimension has equal weight, and each indicator within the dimensions is weighted differently. Households are considered multidimensionally poor if they are deprived in at least one-third of the weighted indicators. Health is a crucial dimension of the MPI and is measured through 2 key indicators: nutrition and child mortality. Nutrition is evaluated by determining whether any household member is malnourished, which reflects inadequate food intake and poor health status. Child mortality is assessed by checking if any child in the household has died within a certain time frame, highlighting access to health care, sanitation, and overall child health. These health measures are considered vital as they affect an individual’s ability to work, learn, and lead a productive life (Alkire and Santos 2010; United Nations Development Programme 2020).

### Organization for Economic Cooperation and Development (OECD) Better Life Index (BLI)

The BLI is designed to measure well-being and quality of life across OECD countries (OECD 2020). This index assesses well-being based on a multidimensional model that includes both objective and subjective measures. The index is structured around 11 key dimensions, including income and wealth, jobs and earnings, housing, health status, education and skills, environmental quality, personal security, work–life balance, social connections, civic engagement, and subjective well-being. Each dimension is designed to capture a specific aspect of well-being that contributes to individuals’ overall life satisfaction and societal progress (Durand 2015; OECD 2020). The BLI uses several indicators to assess health outcomes, including both objective measures (such as life expectancy and mortality rates) and subjective assessments (such as self-reported health). The index also considers inequalities in well-being across population groups, aiming to understand disparities based on age, gender, and socioeconomic status. By integrating these various dimensions, the BLI aims to provide a holistic view of well-being that extends beyond traditional economic indicators such as GDP and to shift the focus to a more inclusive understanding of progress and development in society (OECD 2020).

### United Nations Sustainable Development Goals (SDGs)

The UN SDGs comprise 17 global objectives designed to address various dimensions of sustainability, including economic, social, and environmental aspects, by 2030. Social progress indicators, particularly in health, are crucial to achieving these goals. SDG 3 (“Good Health and Well-being”) specifically aims to ensure healthy lives and promote well-being for all at all ages. It includes the following targets (UNGA and UN Department of Economic and Social Affairs 2015):

- Maternal mortality reduction
- Neonatal and child mortality reduction
- Infectious diseases reduction
- Noncommunicable diseases reduction
- Substance abuse reduction
- Road traffic reduction
- Universal access to sexual and reproductive health care services
- Universal health coverage (UHC)
- Environmental health improvement

Through coordinated efforts, the UN SDGs aim to create a holistic approach to sustainable development, in which social progress and health improvements are foundational to end poverty and inequality, protect the planet, and ensure that all people enjoy health, justice, and prosperity (UNGA and UN Department of Economic and Social Affairs 2015). While specific oral health metrics are absent in the original SDGs (UNGA and UN Department of Economic and Social Affairs 2015), the current WHO strategy and action plan on oral health are aligned with the SDGs, particularly the UHC target of SDG3 (WHO 2024).

While indices for the measurement of social progress have been developed and used for several decades, and although the WHO strategy and action plan on oral health is aligned with the SDGs, oral health indicators are still not comprehensively included as a core component of social progress indices worldwide (see Table 1).

## Why Oral Health Should Be Included in Social Progress Indices

Until now, health markers for social progress were mainly centered around items such as life expectancy, mortality related to infectious and noncommunicable diseases, body height, malnutrition (wasting, stunting, underweight, overweight), fertility (reproductive life span: menarche to menopause), and access to medical care (excluding oral care). While such health markers are inarguably important, they do not capture all relevant dimensions of health and health care that are instrumental for social progress. In particular, and as described in the previous section, previous social progress indices have failed to include oral health. Yet, based on the substantiation provided below, it can be argued that oral health should be included as a key indicator within the health domains of social progress indices:

According to WHO (2024),

Oral health is the state of the mouth, teeth and orofacial structures that enables individuals to perform essential functions such as eating, breathing and speaking, and encompasses psychosocial dimensions such as self-confidence, well-being and the ability to socialize and work without pain, discomfort and embarrassment. Oral health varies over the life course from early life to old age, is integral to general health and supports individuals in participating in society and achieving their potential. . . . Oral diseases disproportionately affect the most vulnerable and disadvantaged populations. People of low socioeconomic status carry a higher burden of oral diseases and this association remains across the life course, from early childhood to older age, and regardless of the country’s overall income level.

As highlighted by the Lancet Oral Health Series, oral conditions have substantial effects on people in terms of causing pain, sepsis, reduced quality of life, lost school days, family disruption, and decreased work productivity (Peres et al. 2019; Watt et al. 2019). Oral conditions share common risk factors with other noncommunicable diseases (NCDs), including free sugar consumption, tobacco and alcohol consumption, and the wider social and commercial determinants of health (Peres et al. 2019; Watt et al. 2019). Oral conditions are correlated with many other health conditions (Seitz et al. 2019; Botelho et al. 2022). Besides pathways through common risk factors, evidence also suggests causal effects of oral conditions on other health conditions such as diabetes, cardiovascular diseases, or depression (D’Aiuto et al. 2018; Matsuyama et al. 2021; Matsuyama et al. 2023).

The costs of oral conditions for individuals and society are considerable (Listl et al. 2015; Righolt et al. 2018; Jevdjevic and Listl 2025). The total worldwide economic impacts of oral conditions were US$710B in 2019, of which US$387B was due to treatment costs and US$323B was due to productivity losses for the 5 main oral conditions (Jevdjevic and Listl 2025). In EU countries, the treatment costs for dental diseases were shown to be the third highest after treatment costs for diabetes and cardiovascular diseases (Listl et al. 2019). Limited oral health coverage has been identified as a key driver of financial hardship (i.e., catastrophic health expenditures) (Thomson et al. 2019; Listl et al. 2021; Proaño et al. 2024).

Hence, oral health is closely intertwined with the fundamental principles of social progress. Drawing from Estes et al. (2023), the definition of social progress can be adapted to oral health as shown in Table 2. Based on these considerations, the Figure summarizes the key characteristics of oral health that qualify it as a pertinent marker of social progress: on the one hand (see circles in the outer layer), oral health is determined by a broad and multifaceted range of factors that reflect (1) social and commercial determinants, (2) health systems determinants, and (3) behavioral and biological determinants. This corresponds to the means required for realizing fuller levels of individual and collective oral health (also see Table 2). On the other hand (see inner core), oral health substantiates considerable impacts on individuals and societies in terms of (1) people’s quality of life, (2) social participation, (3) educational attainment, (4) productivity and income generation, (5) general health, and (6) treatment costs. Thereby, oral health affects individuals and society throughout the entire life course. This corresponds to the ends that result from individual and collective oral health (also see Table 2).

**Table 2.** Social Progress in and through Oral Health.

Oral health is closely intertwined with the fundamental principles of social progress. Drawing from Estes et al. (2023), the definition of social progress can be adapted to oral health as follows: • Social progress entails progressive advancements over time in the capacity of nations to satisfy at least the basic oral health needs of their populations. • Social progress embraces both the means required for realizing fuller levels of individual and collective oral health (through addressing social, commercial, health systems, behavioral and biological determinants) as well as the ends that are to result from that level of individual and collective oral health (quality of life and general health, social participation, educational attainment, productivity and income generation, and reasonable treatment costs). • Social progress in and through oral health is both an intrinsic and instrumental value in social development and, as such, reflects processes and outcomes associated with positive changes that take place on an intentional basis in the lives of people over time.

**Figure.**
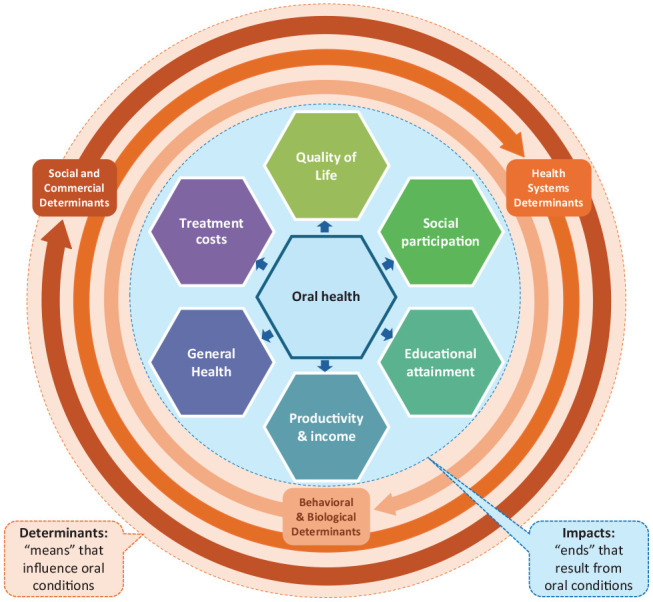
Multifaceted determinants and impacts of oral conditions.

Moreover, the unique characteristics of oral health portray key relevant aspects compared with other health markers of social progress. The advantages of using oral health as marker include (but are not limited to) the following:

- **Embodiment of physical and psychosocial dimensions of health:** While previous health markers for social progress have primarily focussed on physical health dimensions, oral health not only represents a sensible marker for important physical but also for essential psychosocial dimensions of health. In addition to key physical dimensions (eating, breathing, speaking), oral health is closely intertwined with key psychosocial functions such as self-confidence, well-being, and the ability to socialize and work without discomfort or embarrassment (WHO 2024). Oral health signifies a fundamental feature of personal identity, and its strong susceptibility to the broader social determinants is amply documented (Peres et al. 2019; Watt et al. 2019).
- **Relevance throughout the entire life course:** Previous health markers such as body height and childhood or maternal mortality have reflected effects in particular life phases but not health effects throughout the entire life course. For example, body height is largely and accumulatively determined in the first 2 decades of people’s life. Contrarily, people’s oral health status reflects effects that are accumulated throughout the entire life course, that is, from early life to old age (Heilmann et al. 2015). As many people keep some or all of their natural teeth while others become fully edentate throughout life, oral health (particularly people’s number of natural teeth) captures the full load of life-course exposures in a previously absent level of granularity. In addition, oral conditions in early life can be considered as a meaningful predictor for general health issues later in life. Previous evidence suggests that children with greater caries experience are more likely to have poorer self-rated general health by midlife (Ruiz et al. 2023). However, the exact mechanisms through which childhood oral health might be linked with compromised physical health decades later still require further examination.
- **Differential recognition of the impacts of malnutrition:** While previous health markers for malnutrition have primarily focused on undernutrition (wasting, stunting, underweight) or excessive caloric intake (overweight, obesity, and related NCDs), oral health is particularly susceptible to the intake of free sugars, which are central in the etiology of dental caries. As such, oral health marks the effects of sugar consumption (and its underlying commercial determinants) more distinctively than previous health items. In addition, poor oral health is a determinant of malnutrition as it detrimentally affects the ability to eat.
- **Distinctive information on health systems performance:** Previous indices have usually considered generic items for unmet medical needs. Given the enormous limitations in oral health coverage as well as related barriers in affordability and access to essential oral health care (including in countries with high and lower incomes), information on unmet oral health needs provides indispensable insights into health systems performance. This is also corroborated by the European Commission’s recent inclusion of “unmet dental needs” as a new indicator in the EU-SPI 2.0 (European Commission 2024).
- **Good measurability of key oral health information:** Oral health metrics such as the number of teeth or self-reported global oral health, although not yet regularly collected for social progress indices, have shown good measurement properties (see the next section for details). While the (non-oral) health metrics in existing social progress indices also have good measurement properties in general, they are not without limitations. For example, the reliability of mortality information is dependent on the availability and accuracy of diagnostic information and/or death certificates. Limitations also apply to measuring fertility, as self-reports can be biased and clinical observation is complex. Similar difficulties apply to caloric intake or mental health. As such, it is plausible to consider the measurement properties of oral health indicators at least as noninferior to existing social progress indicators.

Hence, oral health is a plausible key indicator for inclusion in the health domains of social progress indices.

## Operationalizing Oral Health as a Marker of Social Progress

A number of initiatives have been working toward harmonization and prioritization of oral health measures, including the EU ADVOCATE consortium, the ICHOM-FDI initiative, and the WHO Oral Health Programme (in line with the UN SDGs; see above). Given the previous dearth and enormous need for more comprehensive oral health monitoring, it seems pertinent that such initiatives have so far been aiming at relatively long lists with detailed oral health items. However, it seems unlikely that all items of such long measurement lists are taken forward as oral health metrics in generic frameworks to monitor social progress.

Currently existing social progress indices already include multifaceted dimensions and many measurement items. Adding new items may not be possible unless the shortening of existing items can be justified. Any newly proposed item thus needs be pertinent and practical. Co-design and consenting oral health indicators together with all relevant stakeholders could facilitate operationalization of suitable items for inclusion in the health domains of social progress indices. Tentatively, such deliberations could draw from oral health metrics as shown in Table 3 and the following aspects (see Table 3):

- **Measurement object:** Oral health involves physical and perceived dimensions. In addition, access to oral care is important to meet oral health needs. The importance of various psychosocial dimensions of oral health has already been highlighted above. As for physical dimensions and according to WHO (2024), “oral diseases encompass a range of diseases and conditions that include dental caries, periodontal (gum) disease, tooth loss, oral cancer, oro-dental trauma, noma and birth defects such as cleft lip and palate.” Because any metric for social progress needs to be pertinent and practical, reducing complexity is key when defining the measurement object. To this end, the measurement objects could tentatively be defined to capture (1) physical oral health as the number of natural teeth, (2) perceived oral health by means of a global oral health rating, and (3) unmet oral health needs. A rationale for the first object could be that the loss of natural teeth results primarily from untreated caries and periodontitis, whereby these 3 conditions together represent the most common oral conditions. These metrics are comparable to health-related items found in existing social progress indices. Self-reported oral health aligns well with self-reported health (e.g., OECD Better Life Index). The number of teeth shares similarities with prevalence metrics for non-communicable diseases and expressing (tooth) mortality. One could also argue that the number of teeth is both an indicator of access to oral health care (the means required for securing good oral health) and of the ends that result from individual and collective oral health. While tooth loss usually becomes more prevalent with increasing age and might be a particularly suitable metric for older adults, perceived oral health might be more relevant among (younger) adults.
- **Assessment method:** While perceived oral health and unmet oral health needs are plausibly measured by means of self-reported information, various alternatives may exist for the assessment of physical oral health. In addition to clinical examination, previous literature has shown that the number of natural teeth can also be identified through self-reports (Sekundo et al. 2019) or remote assessment of images captured by mobile phones (Gurgel-Juarez et al. 2022).
- **Instrument:** The choice of suitable oral health metrics should satisfy validity, reliability, responsiveness, and interpretability in line with the COSMIN Taxonomy of Measurement Properties (Mokkink et al. 2010). The validity of a self-reported instrument to measure the number of teeth has been demonstrated among Europeans aged 50+ y (Sekundo et al. 2019). Locker’s global oral health item has been shown to be a valid instrument among young middle-aged adults in Australia and New Zealand (Thomson et al. 2012). The validity for other populations requires further consideration. This also applies to the “unmet dental needs” instrument as recently included in the EU-SPI 2.0 (European Commission 2024).

**Table 3.** Tentative Metrics to Operationalize Oral Health as a Marker of Social Progress.

Measurement Object	Physical Oral Health: Number of Natural Teeth	Perceived Oral Health: Global Oral Health Rating	Lack of Access to Oral Care: Unmet Oral Health Needs
**Assessment Method**	**Self-Report** [Alternatives: Clinical Examination or Remote Assessment of Mobile Phone Photos]	**Self-Report**	**Self-Report**
**Instrument**	**Q1: *“Do you still have ALL your natural teeth (except wisdom teeth)?”*** [Note: normally a person has 28 teeth + 4 wisdom teeth. We are NOT interested in wisdom teeth] → Binary response options: “***Yes/No.***” Additional item, if the response to the question above was “*No*”: **Q2: *“About how many natural teeth are you missing?*** → Response options: natural number between 1 and 28. (Source: Sekundo et al. 2019)	**Q: “*How would you describe the health of your teeth or mouth?”*** Ordinal response options: • ***Excellent*** • ***Very good*** • ***Good*** • ***Fair*** • ***Poor*** (Source: Thomson et al. 2012)	**Q1: *“Was there any time during the last 12 months when you really needed a dental examination or treatment for yourself?”*** → Binary response options: “***“Yes/No.***” Additional item, if the response to the question above was “Yes”: Q2: ***“Did you have a dental examination or treatment each time you really needed?”*** → Binary response options: “***Yes/No.***” (Sources: European Commission 2024; Eurostat 2021)

As many of the existing social progress indices extract information from (inter-)national data sources that do not yet entail comprehensive oral health information, the question about suitable sources of oral health data to feed into social progress indices arises. While a more structural and consolidated inclusion of oral health items in routinely collected data sources that inform social progress indices would be very much desirable for the future, the provisional use of existing multicountry data sources that contain some albeit limited oral health information such as WHO STEPS (Riley et al. 2016), the GBD Study (Murray et al. 2012), EU-SILC (Eurostat 2021), or SHARE and related studies (Börsch-Supan et al. 2005) would seem plausible for the short run. Note that vast limitations have been reported for existing oral health epidemiological data in terms of inadequate and incomplete reporting of the measurement of oral conditions as well as a lack of consistency and comparability with other health conditions (Bernabé et al. 2025). For the longer run, consenting a worldwide set of key oral health indicators for monitoring social progress could promote the worldwide harmonized use of items such as the number of teeth (see above) in national surveys. If oral health should become an integral part of social progress indices, it would be logical that the collection of oral health data also becomes a more comprehensive component of broad multidisciplinary and multinational data sources that feed into the routine monitoring of social progress. Aspects of feasibility and affordability of oral health metrics in regularly collected questionnaires across countries have been discussed in previous literature (Allison et al. 2023). More generally, the global inclusion of oral health in social progress indices would strongly stimulate improvements in the comprehensiveness of routine (inter-)national oral health data.

## Now What?

Social progress in and through oral health is both an intrinsic and instrumental value in social development. Hence, oral health is a plausible key indicator for inclusion in the health domains of social progress indices. To optimize people’s life and well-being, concise oral health metrics such as people’s number of teeth, a global self-reported oral health item, or unmet oral health needs can provide highly pertinent and practical markers of social progress. One could argue that the number of teeth represents both an indicator of access to oral health care (the means required for securing good oral health) and of the ends that result from individual and collective oral health. WHO’s global strategy and action plan on oral health 2023–2030 offers a unique window of opportunity to prioritize suitable oral health metrics and to enhance the uptake of oral health as a routine marker of social progress. Future research is encouraged to demonstrate the operationalization of oral health metrics for inclusion in holistic frameworks for social progress. Oral health offers vast opportunities to advance social progress and human development.

## Author Contributions

S. Listl, T. Bärnighausen, contributed to conception, design, data analysis and interpretation, drafted and critically revised the manuscript. All authors gave final approval and agree to be accountable for all aspects of the work.
